# A Famous Poem

**Published:** 1884-07

**Authors:** 


### A FAMOUS POEM.

SOME of the verses by which Mrs. Barbauld is best known did not appear until after her death, and amongst these is what may be spoken of as a celebrated stanza from her poem on "Life." It is frequently quoted, as it may very well be, as if it were the piece completed in itself. Perhaps our readers will be glad to see it entire.

LIFE.

"Life! I know not what thou art,
But know that thou and I must part:
And when, or how, or where we met,
I own to me's a secret yet.
But this I know, when thou art fled,
Where'er they lay these limbs, this head,
No clod so valueless shall be
As all that then remains of me.
O whither, whither dost thou fly,
Where bend unseen thy trackless course,
And in this strange divorce,
Ah, tell where I must seek this compound, I?
To the vast ocean of empyreal flame,
From whence thy essence came,
Dost thou thy flight pursue, when freed
From matter's base encumb'ring weed?
Or dost thou, hid from sight,
Wait, like some spell bound knight,
Through blank oblivion's years the appointed hour,
To break thy trance and reassume thy power?
Or canst thou without thought or feeling be?
Oh, say what art thou when no more thou'rt thee?"

But it is the last verse which has been so repeatedly quoted. Indeed, there is a delightful freshness in its expression, and a bright and animated hope. They are probably among the very last lines which fell from her pen.

"Life! we've been long together,
Through pleasant and through cloudy weather;
'Tis hard to part when friends are dear,
Perhaps 'twill cost a sigh, a tear;
Then steal away, give little warning,
Choose thine own time;
Say not Good night, but in some brighter clime
Bid me Good morning!"

It was Crabbe Robinson who gave the volume in which these verses were published to Miss Wordsworth, the sister of the poet. When Wordsworth next met Robinson he said to him, "Repeat me that stanza by Mrs. Barbauld." Robinson did so. Then he requested him to repeat it again, until he learned it by heart. Then he walked up and down the sitting-room at Rydal repeating it himself, and ended by muttering, "I am not in the habit of grudging people their good things, but I wish I had written those lines!"—*Leisure Hour.*

